# Health managers and community representatives' views of a system-wide intervention to strengthen public healthcare in the Free State, South Africa

**DOI:** 10.4314/ahs.v23i1.79

**Published:** 2023-03

**Authors:** Benjamin Malakoane, Perpetual Chikobvu, Christo Heunis, Gladys Kigozi, Willem Kruger

**Affiliations:** 1 Department of Community Health, University of the Free State, PO Box 339, Bloemfontein; 2 Centre for Health Systems Research & Development, University of the Free State, PO Box 339, Bloemfontein

**Keywords:** Public health managers, district health system, community representatives, leadership and governance, health system strengthening, integration

## Abstract

**Background:**

A system-wide health system strengthening (HSS) initiative, the Health Systems Governance and Accountability (HSGA) intervention, was developed, translated to policy, and implemented in the Free State province. This study assessed health managers (HMs) and community representatives' (CRs) views of the intervention and whether it improved integration and performance.

**Method:**

A questionnaire survey among 147 HMs and 78 CRs and 14 focus group discussions (FGDs) with a mean of 10.3 participants and a total of 102 HMs and 42 CRs, were conducted. The questionnaire and FGD data were descriptively and thematically analysed to triangulate findings.

**Results:**

Many HMs (44%) mostly positioned at the operational levels indicated that implementation of the HSGA intervention did contribute to integration of health services. Most CRs (54%) believed that communities were actively involved in the intervention. However, both the self-administered questionnaire and the FGD data evidenced lack of policy awareness among, especially, operational-level HMs.

**Conclusion:**

From the perspectives of HMs and CRs, the implementation of the intervention was viewed as a step forward in strengthening public healthcare to respond to system deficiencies in the Free State province. Earlier engagement of especially operational-level HMs during reforms may be beneficial in successfully implementing HSS interventions.

## Introduction

Since 1994, several health policies that focus explicitly on alleviation of inequity in healthcare service provision have been implemented in South Africa^1^–^3^. These included the introduction of free-health policies, the district health system, and health sector reforms such as prioritisation of primary health care (PHC), in itself a social justice philosophy^4^. PHC re-engineering in South Africa aims to improve community health through ward-based PHC outreach teams (WBPHCOTs), school health services and district clinicals specialist teams (DCSTs)^5^–^6^. Other relatively recent reforms are the establishment of ‘Ideal clinics,’ defined as clinics that open on time and have staff, infection control measures and security to protect the staff and patients^7^ and a ‘One patient-One File’ system. The net effect of these reforms was more equal distribution of public health resources, as well as greater access to services for previously deprived groups^2^. However, stark policy-implementation gaps – fuelled by inefficient processes or systems such as limited financial resource allocations and delegations, top-down directives, and ineffective supervision and performance management systems – frequently occurred, and there may have been an overall lack of systems thinking^8^–^9^.

Many international health policies recognise the World Health Organization's (2008)^10^ vision that communities should be involved in shaping PHC services^11^. South Africa's National Health Act (No. 63 of 2003)^12^ requires the establishment of clinic and community health centre (CHC) committees with functions prescribed in the provincial legislation in question. A representative hospital board for each central hospital (such as Universitas Hospital in the Free State province) with functions prescribed by the national Minister of Health must be in existence. The relevant Member of the Executive Council (MEC) at the provincial level must appoint a representative board for each public health establishment classified as a hospital or for each group of such establishments and prescribe the functions and procedures for meetings of these boards. Although community participation also takes many other forms such as volunteerism by non-governmental organisations (NGOs) and faith-based organisations (FBOs) in clinic data capturing and lay counselling services, it is especially through their participation in clinic and CHC committees and advocacy in hospital boards that communities can contribute to identifying the health system's weaknesses and help to refine necessary changes in the organisational workings of public health systems at the grassroots level.

The health and wellbeing of South Africans – the majority of whom are dependent on government services^13^ - remain plagued by a relentless burden of infectious and noncommunicable diseases^14^–^15^, persisting social disparities, and inadequate human resources for health^1^–^2^,^17^ to provide care for a growing population with a rising tide of refugees and economic migrants^18^. Despite the fact the public health system has been transformed into an integrated, comprehensive national service, failures in leadership, stewardship and management have led to inadequate implementation of good policies^19^. It is generally accepted that public health services in South Africa are not always of sufficient quality to be effective^20^. External factors such as political preferences for the appointment of public health managers (HMs) have been reported to influence the employment of public health professionals^21^. Resulting management skill deficits have a detrimental effect on the quality of public health services. Shortages and high migration of health professionals^1^–^2^;^17^;^20^;^22^, as well as wide inequality^23^–^25^ and unemployment^26^–^28^, further limit the capacity of the public health sector to meet the health needs of the nation.

The international COVID-19 outbreak has emphasised the need to strengthen public health systems^29^ and to adopt systems thinking in doing so^30^. The idea of adopting a system or ‘whole-system’ approach to understand and implement public health system strengthening (HSS) interventions has gained traction in modern times, especially among health policy-makers^31^–^33^. ‘Whole-system’ designs and methodologies are also increasingly applied in health systems research^34^–^38^. A ‘whole-system’ approach inculcates a way of thinking that considers the importance of linkages and interdependencies between the components of a healthcare system^39^–^40^. Systems thinking is also useful to assess the effectiveness of public policy implementation and service delivery^41^–^42^.

In the current setting, initial understanding of the deficiencies of the public health services provided by the Free State Department of Health (FSDoH) was based on a multi-method situation appraisal^19^. A system-wide HSS initiative, the Health Systems Governance and Accountability (HSGA) intervention, was developed, translated to policy, and implemented. The HSGA intervention and its formalisation into an official policy, the HSGA policy^43^, was designed to address fragmentation and improve public health service delivery in the Free State by the provincial health department in collaboration with its stakeholders. The considered intervention model was an integrated approach to health service delivery in the Free State province starting from the community up through all levels to the central health services. It was designed to improve health system performance by effectively supporting the interaction between disease-specific programmes, complemented by a precise routine assessment method, i.e., the BSC performance-monitoring tool^44^–^45^. This study aimed to assess HMs and CRs' views on whether public health service integration had indeed been enhanced and if the integration of the roles and responsibilities of HMs and CRs, as well as attempts to enhance the Health Information Management System, had improved ‘whole-system’ operations.

## Methods

### Design, population and sampling

A cross-sectional survey including, firstly, an anonymous self-administered questionnaire involving 147 HMs and 78 CRs and, secondly, 14 focus group discussions (FGDs) with 102 HMs and 42 CRs was conducted in the 2017/18 financial year. The mean number of participants per FGD was 10.3. In the case of both the HMs and the CRs convenience sampling applied in as far as only those respondents who responded to invitations to sessions to complete the self-administered questionnaire, and conduct the FGDs, were included. The participating HMs (n=102; 70.83%) mostly worked at the facility (clinic/CHC or hospital) level where they were responsible for operational-level programmes. In the current study, members of the hospital boards and clinic and CHC committees whose views on an intervention to strengthen public healthcare in the Free State were sought, are referred to as community representatives (CRs). As governors of these facilities, they were well placed and sufficiently informed to report on different systems aspects. The CRs are entitled to access any public health information from clinic operational managers or hospital CEOs.

### Data collection

Containing both structured and open-ended questions, the self-administered questionnaire elicited HMs and CRs' views on the effectiveness of the HSGA implementation in improving ‘whole-system’ performance in respect to seven goals of the health department, namely, to improve 1) leadership/governance, 2) financial management, 3) workforce management, 4) PHC re-engineering, 5) infrastructure management, 6) the Health Information Management System; and 7) referral and ‘whole-system’ interventions. The FGDs were conducted by research assistants experienced in this technique to supplement the quantitative data obtained through the self-administered questionnaire with a more in-depth qualitative understanding of whether and how public health service integration had been achieved and had, or had not, improved ‘whole-system’ operations. All sessions were audiotaped with the permission of the participants and transcribed verbatim. At the start of the group session, participants completed an attendance register and an informed consent form. A protocol and focus group guide were used to introduce the study and the focus group questions. The research assistant facilitated the group discussions to keep the participants focused on the topics of interest. The average length of the group discussions was 60 minutes. During the FGDs the participants could also revert to their home languages, i.e., mostly Sesotho and Afrikaans, to better express their views.

### Data analysis

The self-administered questionnaire data were descriptively analysed using STATA 12^46^. The FGD data were transcribed and analysed for emerging issues using NVivo 9^47^. However, the FGD data analysis, interpretation and reflection were continuous and already commenced during discussions and transcribing.

### Ethical considerations

Both the self-administered questionnaires and the FGDs were completed in private conference rooms or halls at the district hospitals. Participation in the study was voluntary. As the questionnaire was completed anonymously and numbers were allocated as identifiers during the FGDs, little or no risk were associated with participation in the study. The study participants were not paid for and did not have to pay to participate in the study. All participants signed an informed consent form. The study was approved by the Health Sciences Research Ethics Committee of the University of the Free State (Referral number HSREC 11/2016).

### Findings

The findings are presented according to the aforementioned departmental goals.

### Leadership/governance

Table 1 presents the self-administered questionnaire findings on HMs and CRs' views on achieving the leadership/governance goal. In respect to whether health sector reforms were implemented, a large proportion of the HMs (n=70; 47.62%) stated that they ‘did not know.’ Contrarily, most CRs (n=46; 58.97%) opined that health sector reforms were indeed taking place. Regarding whether district health plans were being implemented, most HMs (n=87; 59.18%) agreed that these plans were implemented, while almost a third (n=43; 29.25%) ‘did not know.’ Whether hospital service delivery plans were implemented, the largest proportion of the HMs (n=72; 48.98%) ‘did not know,’ while about four in every 10 (n=61; 41.50%) indicated that the plans were indeed being implemented. Whether clinic management structures were in place and functional, the largest proportion of more than four in every 10 HMs (n=65; 44.22%) affirmed the existence and functionality of these structures. However, just more than a third (n=50; 34.01%) ‘did not know.’ Regarding their views of the corporate office's influence on district health plans (n=107; 72.79) and hospital health plans (n=111; 75.51), about three-quarters of the HMs ‘did not know.’

**Table 1:** Views related to Goal 1 Leadership/governance

		Yes n (%)	No n (%)	DNK n (%)
**HMs** **(n=147)**	Health sector reforms implemented	67 (45.58)	10 (6.80)	70 (47.62)
	District health plans implemented	87 (59.18)	17 (11.56)	43 (29.25)
	Hospitals service delivery plans implemented	61 (41.50)	14 (9.52)	72 (48.98)
	Clinic management structures in place and functional	65 (44.22)	29 (19.73)	50 (34.01)
	Corporate office influenced district health plans	33 (22.45)	7 (4.76)	107 (72.79)
	Corporate office influenced hospital health plans	28 (19.05)	8 (5.44)	111 (75.51)

**CRs** **(n=78)**	Health sector reforms were implemented	46 (58.97)	14 (17.95)	18 (23.08)

HMs, health managers; CRs, community representatives, DNK, do not know

The self-administered questionnaire findings corroborated the views expressed by the HMs in the FGDs, during which a recurring emerging theme was that plans from higher levels were not carried through:

“*Things are not seen to the end. There are good things that were started like I think it was after 2015/2016, principals from the province came down to us wanting to know what we needed, and we developed action plans. We were promised things that were never done. It was a good initiative, but it was never seen through*”

[Facility Operational Manager].

The CRs expressed similar views during the FGDs:

“*The problem is that the Department would come with programmes but will not sustain them*.”

“*Get the resources in place and then come back to tick the box to say why is there no improvement or why is there only small improvement, while you have all your equipment, all your resources, the ambulance system in place, the forms are there*.”

“*The policies are very good on paper, we read them, we hear about them, but when it comes to the implementation part of it, it just doesn't happen*.”

### Financial management

Table 2 depicts the HMs and CRs' views on achieving the financial management goal as discerned through the self-administered questionnaire. The largest proportions of both the HMs (n=57; 38.7%) and CRs (n=22; 28.21%) agreed that all hospitals charged fees for services as required. However, most of the HMs indicated that they ‘did not know’ whether records of accounting procedures were in place (n=82; 55.78%), periodic audits were conducted (n=91; 61.90%), monthly financial reports were delivered (n=85; 57.82%), and whether expenditure reports (n=70; 47.62%), revenue reports (n=71; 48.30%), records of accounting procedures (n=77; 52.38%), periodic audit reports (n=84; 57.14%) and monthly financial reports (n=75; 51.02%) were in use. A prominent theme emerging from the FGDs with both the HMs and CRs was constant resource scarcity:

**Table 2:** Views related to Goal 2 Financial management

		All	Most	Some	None	DNK
		n (%)	n (%)	n (%)	n (%)	n (%)
**HMs** **(n =147)**	Hospitals charged fees for services	57 (38.78)	19 (12.93)	31 (21.09)	9 (6.12)	31 (21.09)
	Records of accounting procedures in place	37 (25.17)	15 (10.20)	6 (4.08)	7 (4.76)	82 (55.78)
	Periodic audits conducted	31 (21.09)	9 (6.12)	8 (5.44)	8 (5.44)	91 (61.90)
	Monthly financial reports delivered	41 (27.89)	11 (7.48)	4 (2.72)	6 (4.08)	85 (57.82)
	Expenditure reports in use	44 (29.93)	17 (11.56)	10 (6.80)	6 (4.08)	70 (47.62)
	Revenue reports in use	43 (29.25)	17 (11.56)	11 (7.48)	5 (3.40)	71 (48.30)
	Record of accounting procedures in use	33 (22.45)	21 (14.29)	11 (7.48)	5 (3.40)	77 (52.38)
	Periodic audit reports in use	32 (21.77)	8 (5.44)	16 (10.88)	7 (4.76)	84 (57.14)
	Monthly financial reports in use	46 (31.29)	13 (8.84)	8 (5.44)	5 (3.40)	75 (51.02)

**CRs** **(n=78)**	Hospitals charged fees for services	22 (28.21)	11 (14.10)	17 (21.79)	16 (20.51)	12 (15.38)

CR, community representative; DNK, do not know; HM, health manager; n, number

“*We do not have enough resources for the implementation. If the resources were there, it would be easier for the model to work. One may say that we should push ourselves for the model to work, but you cannot push yourself if you do not have resources*” [Unit leader].

“*Our clinic is in town and our building belongs to the municipality. There is a very small space for consultation. When you go for medication at the pharmacy it is far. You have to walk a distance and there are stairs which is a problem for old people and the disabled*” [CR].

### Workforce management

Table 3 portrays the HMs and CRs' views on achievement of the workforce management goal. The largest proportion of HMs indicated that only ‘some’ district staff had HR functions such as job descriptions (n=53; 36.30%), training (n=59; 40.41%) and career (n=61; 41.78%) plans, and staff assessment (n=62; 42.47%) and rotation (n=67; 45.89%) systems. The largest proportion of HMs also indicated that only ‘some’ hospital staff had job descriptions (n=63; 43.15%), training (n=67; 45.89%) and career (n=70; 47.95%) plans, and staff assessment (n=73; 50.00%) and rotation (n=74; 50.68%) systems. A common theme during the FGDs was workforce or human resource (HR) scarcity. One HM stated:

**Table 3:** Views related to Goal 3 Workforce management

		All staff	Most staff	Some staff	No staff	DNK
		n (%)	n (%)	n (%)	n (%)	n (%)
**HMs** **(n=146)**	Districts had job descriptions	21 (14.38)	40 (27.40)	53 (36.30)	8 (5.48)	24 (16.44)
	Districts had training plans	14 (9.59)	38 (26.03)	59 (40.41)	11 (7.53)	24 (16.44)
	Districts had career plans	11 (7.53)	35 (23.97)	61 (41.78)	14 (9.59)	25 (17.12)
	Districts had staff assessment systems	15 (10.27)	37 (25.34)	62 (42.47)	10 (6.85)	22 (15.07)
	Districts had staff rotation systems	11 (7.53)	31 (21.23)	67 (45.89)	12 (8.22)	25 (17.12)
	Hospitals had job descriptions	10 (6.85)	39 (26.71)	63 (43.15)	4 (2.74)	30 (20.55)
	Hospitals had training plans	7 (4.79)	37 (25.34)	67 (45.89)	8 (5.48)	27 (18.49)
	Hospitals had career plans	7 (4.79)	32 (21.92)	70 (47.95)	9 (6.16)	28 (19.18)
	Hospitals had staff assessment	6 (4.11)	35 (23.97)	73 (50.00)	6 (4.11)	26 (17.81)
	Hospitals had staff rotation systems	5 (3.42)	37 (25.34)	74 (50.68)	4 (2.74)	26 (17.81)

				**Yes**	**No**	**DNK**
				**n (%)**	**n (%)**	**n (%)**

	Hospitals had up-to-date staff status reports			33 (22.60)	6 (4.11)	107 (73.29)
	Performance agreements of all the staff in HM's institution up-to-date			59 (40.41)	21 (14.38)	66 (45.21)
	Morale of staff in HM's institution is assessed			48 (32.88)	37 (25.34)	61 (41.78)

		**All staff**	**Most staff**	**Some staff**	**No staff**	**DNK**
		**n (%)**	**n (%)**	**n (%)**	**n (%)**	**n (%)**

**CRs** **(n=78)**	Morale of the staff is always checked	26 (33.33)	13 (16.67)	11 (14.10)	13 (16.67)	15 (19.23)

CR, community representative; DNK, do not know; HM, health manager; n, number

“*What happens at this hospital is that you have to receive that patient, but because you don't have such people with such skills, the anaesthetist, what happens to that patient? Complications come, now preventable medico-legal hazards or medical litigations will occur. On the other hand, working relationships with* [HMs] *become strained because now you start fighting*” [Unit leader].

The CRs likewise voiced concerns about human resource (HR) shortages:

“*We have only one assistant pharmacist and when she has personal stuff we have no one to replace her. Sometimes it happens that she is not there and there is only one sister at work. The sister has to consult and go to the pharmacy to dispense medication to the patients, which is almost impossible*.”

Large proportions of the HMs indicated that they ‘did not know’ whether staff roles in performance agreements were up-to-date (n=66; 45.21%) and if the morale of staff was assessed (n=61; 41.78%) in their institutions. Contrarily, the largest proportion of CRs (n=26; 33.33%) indicated that staff morale was indeed assessed.

### PHC re-engineering

Table 4 describes the HMs and CRs' views on re-engineering of and improving access to PHC. Large proportions of the HMs indicated that school health (n=62; 42.76%), outreach (n=63; 43.15%), healthy lifestyle promotion (n=60; 41.10%), family health (n=62; 42.47%), DCST (n=49; 33.56%), and contracted general practitioner (n=57; 39.04%) and ‘development partner’ (n=59; 40.41%) services were at least ‘partially’ integrated into the system.

**Table 4:** Views related to Goal 4 PHC re-engineering

	Views on extent to which PHC services were integrated into system	Completely n (%)	Partially n (%)	Not at all n (%)	DNK n (%)
**HMs (n=146)**	School health teams	42 (28.97)	62 (42.76)	5 (3.45)	36 (24.83)
	Outreach services	42 (28.97)	63 (43.15)	8 (5.48)	33 (22.60)
	Healthy lifestyle promotion	38 (26.03)	60 (41.10)	13 (8.90)	35 (23.97)
	WBPHCOTs	35 (23.97)	62 (42.47)	11 (7.53)	38 (26.03)
	DCST services	32 (21.92)	49 (33.56)	19 (13.01)	46 (31.51)
	Contracted general practitioners	23 (15.75)	57 (39.04)	15 (10.27)	51 (34.93)
	Development partners	39 (26.71)	59 (40.41)	6 (4.11)	42 (28.77)

	**Views whether communities were actively involved in implementation** **of health reforms**		**Yes** **n (%)**	**No** **n (%)**	**DNK** **n (%)**

**CRs (n=78)**	Ideal clinic		56 (71.79)	6 (7.69)	16 (20.51)
	HSGA intervention		42 (53.85)	7 (8.97)	29 (37.18)
	BSC performance-monitoring tool		37 (47.44)	5 (6.41)	36 (46.15)
	One patient-One file		51 (65.38)	3 (3.85)	24 (30.77)

BSC, Balanced Scorecard; CR, community representative; DCST, District Clinical Specialist Team; DNK, do not know; HM, health manager; HSGA, Health System Governance and Accountability; n, number; PHC, primary health care; WBPHCOT, ward-based PHC outreach team

Most CRs (n=56; 71.79%) indicated that communities were ‘actively involved’ in implementing the ‘Ideal clinic,’ defined as a clinic that opens on time and has staff, infection control measures and security to protect the staff and patients^7^. Most CRs also agreed that communities were actively involved in implementing the ‘One Patient-One file’ system, i.e., a single patient identifier and a single file containing all the medical history of an individual patient (n=51; 65.38%). They also mostly agreed that communities were ‘actively involved’ in the implementation of the HSGA intervention (n=42; 53.85%). Just less than half of the CRs (n=37; 47.44%) believed that communities were ‘actively involved’ in implementing the Balanced Scorecard (BSC) performance-monitoring tool.

### Infrastructure management and the Health Information Management System

Table 5 depicts the HMs and CRs' views on, firstly, infrastructure management and availability of equipment and, secondly, the Health Information Management System. Regarding infrastructure management, the largest proportion of HMs (n=68; 46.58%) indicated that they did not participate in meetings on strategic infrastructure planning. Most HMs also stated that availability (n=68; 53.54%) and maintenance (n=85; 58.22%) of equipment at health facilities did not meet expectations. Nearly six in every 10 HMs thought that the lack of infrastructure maintenance (n=87; 59.59%) and unavailability of equipment at health facilities (n=86; 58.90%) had a ‘negative’ influence on public health system performance. HMs frequently raised the lack of maintenance during the FGDs: “*There is absolutely no maintenance. There was this buzz word that was called ‘maintenance hub,’ and we bought into it saying at least if my door is broken, assistance will just be a call away. Or maybe one of the clinics would be having a hub where I could call to say my stuff is broken. But now you have to go on trying to fix stuff like your light bulbs. You are frustrated because you want to use something, but you can't*” [facility Operational Manager]. Equal proportions (n=25; 32.05%) of the CRs agreed or disagreed that they participated in meetings on strategic infrastructure planning. As with most HMs, most CRs also indicated that infrastructure availability (n=45; 57.69%) and maintenance (n=41; 52.56%) at health facilities did not meet expectations. Large proportions of the CRs believed that the lack of infrastructure maintenance had a ‘negative’ (n=27; 34.62%) or a ‘very negative’ (n=17; 21.79%) influence on public health system performance. Even larger proportions thought that the unavailability of equipment at health facilities had a ‘negative’ (n=29; 37.18%) or ‘very negative’ (n=22; 28.20%) influence on the system's performance.

**Table 5:** Views related to Goal 5 Infrastructure management and Goal 6 Health Information Management System

				Yes n (%)	Partially n (%)	No n (%)	DNK n (%)
**Infrastructure** **management**	**HMs** **(n=146)**	Participated in meetings on strategic infrastructure planning		22 (15.07)	22 (15.07)	68 (46.58)	34 (23.29)
		Availability of equipment met expectations		15 (11.81)	31 (24.41)	68 (53.54)	13 (10.24)
		Infrastructure maintenance met expectations		8 (5.48)	19 (13.01)	85 (58.22)	34 (23.29)

			**VN** n (%)	**Neg** n (%)	**SN** n (%)	**NN** n (%)	**DNK** n (%)

		Influence of unavailability of equipment on health service performance	86 (58.90)	17 (11.64)	11 (7.53)	6 (4.11)	26 (17.81)
		Influence of lack of maintenance on health systems performance	87 (59.59)	15 (10.59)	13 (8.90)	2 (1.37)	29 (19.86)
	
				**Yes** n (%)	**Partially** n (%)	**No** n (%)	**DNK** n (%)

	**CRs** **(n=78)**	Participated in meetings on strategic infrastructure planning		25 (32.05)	18 (23.08)	25 (32.05)	10 (12.82)
		Availability of equipment met expectations		17 (21.79)	15 (19.23)	45 (57.69)	1 (1.28)
		Infrastructure maintenance met expectations		20 (25.64)	12 (15.38)	41 (52.56)	5 (6.41)

			**VN** n (%)	**Neg** n (%)	**SN** n (%)	**NN** n (%)	**DNK** n (%)

		Influence of unavailability of equipment on health system performance	22 (28.20)	29 (37.18)	16 (20.51)	4 (5.12)	7 (8.97)
		Influence of lack maintenance on health system performance	17 (21.79)	27 (34.62)	14 (17.95)	4 (5.13)	16 (20.51)

					**Yes** n (%)	**No** n (%)	**DNK** n (%)

**Health** **information** **management** **system**	**HMs** **n (147)**	Whether possible based on the DHIS to indicate five diseases with the highest consultation rates in all districts			78 (53.06)	12 (8.16)	57 (38.78)
		Whether possible based on the DHIS to indicate five diseases with the highest consultation rates in HM's district			80 (54.79)	13 (8.90)	53 (36.30)

			**Always** n (%)	**Mostly** n (%)	**Seldom** n (%)	**Never** n (%)	**DNK** n (%)

		Frequency of statistics form shortages at facilities	11 (7.59)	18 (12.41)	47 (32.41)	26 (17.93)	43 (29.66)
		Frequency of submission of data to the DHIS by facilities	96 (66.21)	7 (4.83)	3 (2.07)	0 (0)	39 (26.90)
		Frequency of analysis of statistics for decisionmaking by facility staff	40 (27.59)	34 (23.45)	24 (16.55)	5 (3.45)	42 (28.97)
		Frequency of feedback reports to facilities by district-level staff	40 (27.59)	29 (20.00)	42 (28.97)	13 (8.97)	5 (3.45)
		Frequency of use of health activity monitoring mechanisms	24 (16.55)	28 (19.31)	36 (24.83)	54 (37.24)	45 (31.03)
		Frequency of submission of reports to the DHIS by traditional healers	6 (4.14)	6 (4.14)	18 (12.41)	5 (3.45)	84 (57.93)
		Frequency of submission of reports to the DHIS by NGOs	27 (18.62)	20 (13.79)	11 (7.59)	17 (11.72)	80 (55.17)
		Frequency of submission of reports to the DHIS by FBOs	8 (5.52)	12 (8.28)	7 (4.83)	24 (16.55)	94 (64.83)
	
					**Yes** n (%)	**No** n (%)	**DNK** n (%)

	**CRs** (n=78)	Whether possible to indicate five diseases with the highest consultation rates in CR's district			50 (64.10)	9 (11.54)	19 (24.36)

				**Always** n (%)	**Mostly** n (%)	**Seldom** n (%)	**Never** n (%)

		Frequency of statistics form shortages at facilities		11 (14.10)	19 (24.36)	25 (32.05)	23 (29.49)
		Frequency of submission of data to the DHIS by facilities		22 (28.21)	15 (19.23)	27 (34.62)	14 (17.95)
		Frequency of analysis of statistics for decision-making by facility staff		19 (24.36)	14 (17.95)	24 (30.77)	21 (26.92)
		Frequency of feedback reports to facilities by district-level staff		25 (32.05)	9 (11.54)	21 (26.92)	23 (29.49)
		Frequency of use of health activity monitoring mechanisms		27 (34.62)	10 (12.82)	18 (23.08)	23 (29.49)
		Frequency of submission of reports to the DHIS by traditional healers		25 (32.05)	13 (16.67)	16 (20.51)	24 (30.77)

CR, community representative; DHIS, District Health Information System; DNK, do not know; FBO, faith-based organisation; HM, health manager; n, number; Neg, negative; NGO, non-governmental organisation; NN, not negative; SN, somewhat negative; VN, very negative

In terms of the Health Information Management System, most HMs agreed that it was possible to indicate the five diseases with the highest consultation rates based on the District Health Information System (DHIS) in all districts (n=78; 53.06%), and in their own district (n=80; 54.79%). Almost a third (n=47; 32.41%) of the HMs believed there were ‘seldom’ and a further almost two in every 10 (n=26; 17.93%) that there were ‘never’ any shortages of health statistics forms at health facilities during the previous 12 months. Nearly two-thirds (n=96; 66.21%) of the HMs believed that health facilities always' submitted data to the DHIS. More than a quarter (n=39; 26.90%) stated that they ‘did not know.’ The largest proportion (n=42; 28.97%) of HMs also stated that they ‘did not know’ how often health facility staff analysed statistics for decision-making. However, according to many HMs, the statistics were ‘always’ (n=40; 27.59%) or ‘mostly’ (n=34; 23.45%) considered for decision-making. Many of the HMs believed that district-level staff ‘always’ (n=40; 27.59%) or ‘mostly’ (n=29; 20.00%) provided feedback to health facilities in response to submitted reports. Again, more than a quarter (n=42; 28.97%) of the HMs said they ‘did not know.’ The largest proportion (n=54; 37.24%) of the HMs also ‘did not know’ how often health activity monitoring mechanisms such as charts and diagrams were used, although relatively large proportions said such mechanisms were ‘mostly’ (n=36; 24.83%) or ‘always’ (n=24; 16.55%) used. The majority of HMs indicated that they ‘did not know’ how often traditional leaders (n=84; 57.93%), NGOs (n=80; 55.17%) and FBOs (n=94; 64.83%) submitted reports to the DHIS. The theme of health information resource scarcity was often raised during the FGDs:

“*We don't even have a photocopy machine through the whole of Mangaung Metro*” [Facility Operational Manager].

“*If you haven't got stationery to work with, everything else falls apart*” [Facility Operational Manager].

Regarding the CRs' views, almost two-thirds (n=50; 64.10%) affirmed that it was possible – based on the DHIS – to indicate which five diseases had the highest consultation rates in their district. The largest proportion (n=25; 32.05%) of CRs thought that health facilities ‘seldom’ experienced shortages of statistics forms. However, the largest proportion of CRs (n=24; 30.77%) was also of the view that health facility staff ‘seldom’ analysed statistics for decision-making. Less than a third of the CRs (n=25; 32.05%) indicated that district-level managers ‘always’ provided feedback to health facilities in response to reports submitted.

### Referral and ‘whole-system’ interventions

Table 6 depicts the HMs and CRs' views on optimising referral and ‘whole-system’ interventions (i.e., the HSGA intervention and the BSC performance-monitoring tool). Most HMs believed that referral processes (n=93; 64.14%), referral notes (n=85; 58.62%) and monitoring of waiting times (n=80; 55.17%) were ‘always’ in place. A large proportion (n=64; 44.14%) also stated that ambulance referral or dispatch systems were ‘always’ in place. However, according to the largest proportion of HMs (n=44; 30.4%), referral feedback reports were ‘seldom’ received. Most of the HMs also ‘did not know’ whether agreements on the referral of patients from traditional leaders (n=83; 57.24%) and NGOs (n=73; 50.34%) were in place. A prominent theme during the FGDs with HMs was that plans and changes were insufficiently communicated to and discussed with lower cadres of HMs and staff:

“*Since 2015, as you see us, we don't have information to say confidently that we know about the model. The exposure I personally had about it was like in a conference setup where you cannot really interact with it because it was just a presentation. However, I think it is a viable model that can benefit many institutions*” [Facility Operational Manager].

**Table 6:** Views related to Goal 7 Referral and whole-system interventions

			Always n (%)	Mostly n (%)	Seldom n (%)	Never n (%)	DNK n (%)
**HMs** **(n=147)**	**Referral**	Processes to refer patients to other facilities in place	93 (64.14)	18 (12.41)	2 (1.38)	0	32 (22.07)
		Referral notes (from a lower to a higher level) in place	85 (58.62)	23 (15.86)	5 (3.45)	1 (0.69)	31 (21.38)
		Referral feedback reports (from higher level back to lower level) in place	33 (22.76)	24 (16.55)	44 (30.34)	15 (10.34)	29 (20.00)
		Ambulance referral/dispatch systems in place	64 (44.14)	41 (28.28)	7 (4.83)	2 (1.38)	31 (21.38)
		Waiting times in healthcare facilities monitored	80 (55.17)	28 (19.31)	5 (3.45)	1 (0.69)	31 (21.38)
		Agreements on referral of patients from traditional healers in place	15 (10.34)	7 (4.83)	6 (4.14)	34 (23.45)	83 (57.24)
		Agreements on referral of patients from NGOs in place	23 (15.86)	19 (13.10)	16 (11.03)	14 (9.66)	73 (50.34)

**CRs** **(n=78)**	**Referral**	Processes to refer patients to other facilities in place	38 (48.72)	10 (12.82)	10 (12.82)	8 (10.26)	12 (15.38)
		Referral notes (from a lower to a higher level) in place	41 (52.56)	10 (12.82)	9 (11.54)	4 (5.13)	14 (17.95)
		Referral feedback reports (from higher level back to lower level) in place	34 (43.59)	14 (17.95)	9 (11.54)	7 (8.97)	14 (17.95)
		Ambulance referral/dispatch systems in place	36 (46.15)	8 (10.26)	15 (19.23)	9 (11.54)	10 (12.82)
		Waiting times in healthcare facilities monitored	33 (42.31)	10 (12.82)	11 (14.10)	11 (14.10)	13 (16.67)
		Agreements on referral of patients from traditional healers and NGOs in place	26 (33.33)	9 (11.54)	10 (12.82)	11 (14.10)	22 (28.21)

					**Yes** n (%)	**No** n (%)	**DNK** n (%)

**HMs** **(n=147)**	**Whole-system** **interventions**	HSGA intervention contributed to integrating health service delivery			65 (44.22)	18 (12.24)	64 (43.54)
		BSC performance-monitoring tool contributed to integrating health service delivery			60 (40.82)	23 (15.65)	64 (43.54)
		HSGA intervention contributed to improving health outcomes			61 (41.50)	17 (11.56)	69 (46.94)
		BSC performance-monitoring tool contributed to improving health outcomes			60 (40.82)	23 (15.65)	64 (43.54)
		HSGA intervention contributed to integrating health service delivery			53 (67.95)	9 (11.54)	16 (20.51)
		BSC performance-monitoring tool contributed to integrating health service delivery			39 (66.10)	7 (11.86)	32 (22.03)
		HSGA intervention contributed to improving health outcomes			38 (64.41)	5 (8.47)	35 (27.12)
		BSC performance-monitoring tool contributed to improving health outcomes			43 (72.88)	4 (6.78)	31 (20.34)

BSC, Balanced Scorecard; CR, community representative; DNK, do not know; HM, health manager; HSGA, Health System Governance and Accountability; n, number; NGO, non-governmental organisation

Regarding the HMs' views on the effects of the implementation of the HSGA intervention and the BSC performance-monitoring tool, large proportions believed that the intervention (n=65; 44.22%) and the tool (n=60; 40.82%) contributed to the integration of health service delivery. Similar proportions of the HMs believed that the HSGA intervention (n=61; 41.50%) and the BSC performance-monitoring tool (n=60; 40.82%) contributed to improving health outcomes. The issue of plans from higher levels not being carried through arose again during the FGDs with both HMs and CRs:

“*When the model was actually introduced formally, the senior management teams at different facility levels were the ones that were exposed to the whole unpacking of the model, but I think cascading down has been an issue*” [Facility Operational Manager].

“*I think the system is very good and the aim of it is excellent, but the implementation is not there*” [CR].

“*This needed to be communicated down to the communities as well so that they understand why they are not referred to their preferred hospital and so that they do not feel dissatisfied that they are not looked after well*” [CR].

The largest proportions of CRs indicated that referral processes (n=38; 48.72%), referral notes (n=41; 52.56%), referral feedback reports (n=34; 43.59%), ambulance referral/dispatch systems (n=36; 45.15%), monitoring of waiting times (n=33; 42.31%) and agreements on referral of patients from traditional healers and NGOs (n=26; 33.3%) were ‘always’ in place. Regarding the CRs' views on the effects of the implementation of the HSGA intervention, most thought that the intervention contributed to integrate health service delivery (n=53; 67.95%) and improve health outcomes (n=38; 64.41%). Likewise, they mostly believed that the BSC performance-monitoring tool contributed to integrate health service delivery (n=39; 66.10%) and improve health outcomes (n=43; 72.88%).

## Discussion

The discussion is again structured according to the seven departmental goals.

### Leadership/governance

In order to operate a provincial health system, district health plans are developed under the direction of the corporate office by the district managers. Their teams comprise of operational managers based in the facilities and the programme managers and coordinators that support them. Thwala et al.^48^ posited that the positioning of the district managers as frontline service delivery ‘stewards,’ and provincial-level managers as ‘overseers,’ places the role of leadership/governance at the core of health service delivery to ensure that the district health system (DHS) works to achieve common goals. Contrary to Kemppainen's^49^ observation that HMs should lead the implementation of plans within their organisations and influence their teams to respond to plans and reforms, including implementation of daily management processes, this was apparently not the case in the Free State. This could partially be attributed to employee attitudes as three of the most significant barriers to managing change include lack of management visibility and support, employee resistance to change and inadequate management skills^50^.

Effective management skills are key to helping organisations through times of change. Applebaum & Wohl^50^, p. 279 wrote that notwithstanding the best efforts of senior healthcare executives, major change initiatives often fail: “Change threatens the very stability and continuity that managers are attempting to control; therefore, change and managers are not natural partners.” According to these authors, even those managers cognisant of the need for change resist parts that they see as “too major, too risky, or too different.” However, successful initiatives to improve systems management and transformation have been reported. In 2019, a case study of a rural district in Mpumalanga province in South Africa described how declines in morbidity and mortality from severe acute malnutrition in young children were achieved following a district HSS approach embedded in supportive policy and processes at the national and provincial levels^38^. Centred on real-time death reporting, analysis and response, the HSS interventions produced three kinds of system-level change: 1) knowledge and use of evidence by providers and managers (‘ways of thinking’), 2) leadership, participation and coordination (‘ways of governing’) and 3) inputs and capacity (‘ways of resourcing’). The study found that ‘whole-system’ approaches and coordinating action at multiple levels, were imperative in building enabling environments at the frontline.

### Financial management

Public hospitals in South Africa are compelled to collect revenue as part of their budget and in line with their revenue collection strategy. While large proportions of both the HMs and CRs agreed that all hospitals charged fees for services, more than half of the HMs indicated that they ‘did not know’ whether financial management procedures and reports were in place. This is concerning since these are day-to-day financial management functions, and HMs are expected to know about them. Therefore, the observed spate of ‘don't know’ was an unexpected response from fiduciaries entrusted to know and enact their mandate and internal functional and financial responsibilities.

Many HMs who lacked knowledge about basic managerial financial functions may have risked managerial abdication of responsibility or accountability as regulated by the Public Finance Management Act (PFMA)^51^ and National Treasury Regulations and Instruction Notes^52^–^53^. Given the importance of financial resources as a driver of change^38^;^54^, these are concerning findings. However, the explanation for the wave of ‘don't know’ answers could again be that the survey may have been conducted too early in the HSGA intervention process. Indeed, implementation of the HSGA model may have contributed to improved financial management over the longer term as in 2016/17 and again in 2017/18 the FSDoH obtained unqualified audit opinions^55^.

### Workforce management

Human resources (HR) have been described as one of the three most imperative health system inputs. The other two important inputs are physical capital and commodities^56^. Provision of quality healthcare services requires a committed and dedicated workforce that can work within an environment conducive to unleashing internal talents or skills. While service industries outside the health sector continuously demonstrate that transformational and service leadership styles are most efficacious in energising HR within organisations, healthcare organisations are often dominated by leaders who practice outmoded transactional types of leadership^57^. The 1997 White Paper for the Transformation of the Health System in South Africa^58^ directed that management authority should be decentralised to the provincial and district levels to allow for a greater degree of autonomy and that HMs should be supported in acquiring the skills required to manage the HR of a decentralised health service.

In the current study, the largest proportion of HMs indicated that only ‘some’ staff in the districts and hospitals had job descriptions, training and career plans, and staff assessment and rotation systems. Large proportions of the HMs indicated that they ‘did not know’ whether staff roles in performance agreements were up-to-date and if staff morale was assessed in their institutions. Basic HR management capability challenges were thus evident among the HMs. A previous study^59^ suggested that poor performance within organisations was directly related to weak or poor leadership, consequently yielding a negative impact on employee morale, performance and service delivery. A survey of American medical directors indicated that competencies related specifically to healthcare and clinical skills proficiencies, were more highly recognised relative to generic administrative or management skills^60^. This negated the fact that some background in management or some specialised training in healthcare management are essential skills for effective management of health services. Therefore, the importance of determining management capabilities and providing appropriate skills training was required as part of an overall management development process to improve policy and procedure implementation and health systems functioning^61^. This is corroborated by the National Department of Health which directs that HMs should be supported in acquiring the competencies required to manage a decentralised health service with context-sensitive workload indicators^62^. The current study's findings suggest that to deal with capacity challenges, HMs in the Free State would particularly benefit from training in leadership, HR and organisational behaviour.

### PHC re-engineering

Former South African Health Minister Aaron Motsoaledi's turnaround strategy considered PHC re-engineering to be the main and foundational pillar of health system reform^63^. A well-functioning DHS is required for effective re-engineering of PHC. This strategy requires strong leadership and greater emphasis on health promotion, prevention, and community participation and empowerment^64^. The current study considered the HMs' views on the extent to which PHC re-engineering services, including school health, outreach, healthy lifestyle promotion (e.g., anti-tobacco, alcohol and substance abuse), family team (WBPHCOT), DCST, contracted general practitioner, and ‘development partner’ services were integrated into the Free State public health system. Large proportions of the HMs thought that such services were only ‘partially’ integrated. This is concerning because PHC is a vital pillar for fundamental health care at the community level and is also considered a cost-effective modality^65^. On the positive side, most CRs affirmed that communities were involved in implementing interventions (e.g., Ideal clinic model, HSGA intervention, BSC performance-monitoring tool and One Patient-One file reform efforts). However, in September 2021, Ritshidze^66^, p. 39 reported that only six out of 22 PHC facilities it monitored in three of the five districts in the Free State had ‘functional’ clinic committees.

Adopting the view that the extensive revisions to the health system represent ‘whole-system’ (as opposed to piecemeal or programmatic) change, Gilson et al. (2017)^67^ enumerated the lessons learnt in transforming the health system in the Western Cape and made recommendations for the successful implementation of such an approach. These included meeting the need for new PHC models oriented to the wider health and social challenges facing populations in the 21^st^ century; development of inter-sectoral partnerships and multiple forms of patient and community engagement; and innovative action to address the health challenges of particularly vulnerable groups and communities. However, recent evidence shows that health committees are not functioning effectively in South Africa^68^. Factors impacting negatively on health committee participation include health committees' unclear roles; committee members' skill deficits; facility managers and ward councillors' negative attitudes; and limited resources, support and recognition^69^.

### Infrastructure management and the Health Information Management System

Infrastructure is considered an integral aspect of quality health service provision and a key pillar supporting the fundamental objective of promoting good standards of care for all patients^70^, p. 5: “This requires scrutiny of every element of the life cycle of a device from the specification of requirements, through evaluation of competing products, decontamination, procurement, introduction, maintenance and quality assurance to disposal and funded plans for equipment replacement.” However, old and poorly maintained healthcare infrastructure is a pervasive problem affecting the delivery of quality healthcare in many parts of South Africa^71^ and the Free State province^72^. In this study, HMs and CRs' views on whether infrastructure availability and maintenance met expectations and the effects thereof on public health system performance were assessed. Most HMs indicated that they did not participate in infrastructure planning meetings, that the infrastructure's availability and maintenance did not meet expectations, and affirmed the ‘negative’ or ‘very negative’ influence that unavailability of equipment and lack of maintenance had on the public health system's performance.

Most of the CRs stated that they at least ‘partially’ participated in infrastructure planning meetings. However, they also mostly agreed with the HMs that the availability and maintenance of infrastructure maintenance did not meet expectations. It is thus clear that the public health infrastructure in the Free State was not congruent with the view that “the quality of patient care and access to services is essentially determined by the quality of infrastructure, quality of training, competence of personnel and efficiency of operational systems”^73^. Ritshidze^66^, p. 16 recently reported that of the 22 clinics in the districts of the Free State it monitored, 27% were in poor condition, 95% needed additional space, 43% did not have enough room in the waiting area, 60% of toilets were in a ‘bad condition,’ and 27% of patients said facilities were ‘dirty’ or ‘very dirty.’

Since the early 1980s, the WHO has emphasised the importance of the Health Information Management System and skills training in implementing an integrated PHC approach^74^. Healthcare should be supported by systems to deliver care that is ‘safe, effective, patient-centred, timely, efficient, and equitable,’ and health information management has a critical role in designing such a system^75^. As more money is spent on health information management, the demand for the cost-effectiveness of healthcare creates new pressures to assess their impact and whether they are achieving “their putative benefits and justifying their costs”^76^, p. 549. Research in the Western Cape province emphasised the need for new forms of monitoring and evaluation that take a ‘whole-system’ perspective: “extending beyond services and programmes to system functions, drawing in a wider range of perspectives and knowledge, and considering not only what but also how health-system change is unfolding”^67^, p. 64.

When the opinions of the HMs and CRs in the Free State were sought on whether it was possible to identify the five diseases with the highest consultation rate in their areas of jurisdiction, both groups mostly affirmed that this was indeed possible. Also positive was that most HMs thought there were ‘never’ any shortages of health statistics forms at health facilities and that health facilities ‘always’ submitted data to the DHIS. Worrying was that the largest proportion of HMs said they ‘did not know’ how often health facility staff analysed statistics for decision-making. According to about half of the HMs, statistics were 'always' or ‘mostly’ considered for decision-making.

The Health Information Management System is a tool intended to enable HCWs at every level to use data for planning, implementation and evaluation. Notable in the current study was that many HMs lacked knowledge or awareness of health information management procedures. Subsequently, the aims of the Health Information Management System and the DHIS to contribute positively to managerial functions of planning, trend analysis, and ensuring quality and efficient healthcare, could not be achieved, and these systems did not serve as a driver of decentralised decision-making^77^.

The DHIS is a platform on which people, processes and technology interact to support the operations and general management to plan, decide, organise and manage the delivery of quality healthcare services^78^. Therefore, buy-in by both top and operational-level managers is essential for adopting and implementing the DHIS as a tool for management. In instances where the DHIS has not been adopted as a strategic imperative, the lack of commitment thereto by management at many levels manifests as an obstacle to the effective implementation of HSS interventions.

Despite wide training on the use of the DHIS, HMs and HCWs were essentially unable to put the data collected to best use for planning and decision-making. It seems that the training of HMs needs to include continuously supporting them and providing oversight on the importance, ability and use of health information^76^. Williamson et al.^74^ recommended that monitoring of the progress of implementation of DHISs must be at three levels of the information system establishment: firstly, at the level of data collection, capturing, validation and reporting; secondly, at the level of data interpretation and presentation; and, thirdly, at the level of information use for decision-making and planning. Appropriate use of DHIS data can transcend good patient care and improve planning, administrative effort, clinical research and strategic information management to improve health system performance^77^;^79^.

Research in several African countries have reported serious challenges in implementing DHIS2. A Malawian study aimed at evaluating how available interface elements influence usability in DHIS2, found that lack of relevant editing features and lack of conformity to the “Keep It Simple, Stupid (KISS) and minimalistic design principle” were challenges negatively affecting the usability of the system^80^. A study to evaluate the quality of reporting of key indicators of childhood malaria during the first four years of DHIS2 implementation in Senegal established that in contrast to public facilities of which 92.7% reported data in the DHIS2 system during the study period, only 15.3% of the private facilities used the reporting system. The quality of reporting for malaria indicators in the Senegal DHIS2 had improved over time and the system was found to be suitable for use to monitor progress in malaria programmes. However, the study recommended that Senegalese health authorities should maintain the focus on broader adoption of DHIS2 reporting by private facilities, the sustainability of district-level data quality reviews, facility-level supervision and feedback mechanisms at all levels of the health system^81^. A Nigerian case study demonstrated the high potential for effective monitoring of maternal and neonatal health using DHIS2^82^. However, coordinated action was needed at multiple levels of the health system to maximize reporting of existing data, rationalise data flow, routinise data quality review, feedback, and supervision, and ongoing maintenance.

### Referral and ‘whole-system’ interventions

The WHO states that “[a]n effective referral system ensures a close relationship between all levels of the health system and helps to ensure people receive the best possible care closest to home” and can help to ensure cost-effective use of hospital facilities, while allowing for timeous access to specialist services^83^. Further to this source, in developing countries many clients seen at outpatient clinics at secondary facilities could be more appropriately looked after at PHC facilities at a lower overall cost to both the client and the health system.

Assessing or monitoring referrals between healthcare services and workers can demonstrate the accomplishments of collective efforts, the balanced use of resources and capabilities through efficient use of network members, and the avoidance of duplication of efforts^84^. In the current study, most HMs stated that referral system processes were ‘always’ in place. A smaller number claimed they ‘did not know.’ Large proportions of HMs believed that that implementing the HSGA and the BSC performance-monitoring tool ‘whole-system’ interventions contributed to integrate health service delivery and improve health outcomes, while most CRs affirmed that the referral systems were ‘always’ or ‘mostly’ in place.

Relatively few studies on initiatives to bring about ‘whole-system’ change in health care in South Africa have been published. In 2014, a case study of factors facilitating early implementation of PHC reform in the Western Cape province, defined ‘whole-system’ interventions as “those that entail system wide changes in goals, service delivery arrangements and relationships between actors, requiring approaches to implementation that go beyond projects or programmes”^35^. The study found that successful implementation of the PHC outreach team strategy was characterised by factors such as a favourable provincial context of a well-established district and sub-district health system and longstanding values in support of PHC, a collective vision for the new strategy, distributed leadership and ownership of the new policy, an implementation strategy that ensured alignment of systems and appropriate sequencing of activities, privileging of community dialogues and local manager participation in the early phases, establishment of special implementation structures to enable feedback and ensure accountability, and an NGO partnership to support implementation.

A limitation of the current study is that its cross-sectional design precludes reporting of the longer-term impact of the concomitant implementation of HSGA intervention and BSC performance-monitoring tool. A strength of the study is that it is the first ‘whole-system’ assessment of a HSS intervention in the Free State province, using more than one technique, which allowed for triangulation of findings. The positionality of the first author as political executive driving the HSS intervention, may be interpreted as limitation as an element of bias may have been inevitable. However, the fact that the researcher was the executive authority of the FSDoH, could also be interpreted as a strength of the study. Indeed, the position held by the first author enabled the scale of the intervention and high level of participation by employees and senior managers. This provided for deeper insights into issues not readily observable to an external observer and conveyed a deeper awareness of multiple problems within the health system.

## Conclusion

Policy-makers and managers of organisational transformation initiatives in healthcare can benefit from periodic assessments of the implementation of HSS interventions. Midcourse assessments of progress and response to evidence of unintended, emergent developments, provide decision-makers with opportunities to reassess and modify their plans. In the current study, HMs and CRs' views of a system-wide intervention to strengthen public healthcare in the Free State, the HSGA model, were studied. The great number of ‘don't know’ responses to questions about leadership/governance, financial management, workforce management, PHC re-engineering, infrastructure and information management, and referral and whole-system interventions, were likely indicators of serious policy-implementation gaps and did not portend well for the state of public healthcare in the Free State. The preponderance of “don't know” answers about issues that fall within the realm of their day-to-day activities - as also outlined in their job descriptions – requires a re-look at what the possible reasons for this apparent apathy and seeming policy-implementation gaps could be. On the positive side, the HMs and CRs mostly thought that the HSGA intervention and the BSC performance-monitoring tool were effective in integrating public health service delivery and improving performance and creating a good platform for achieving the desired outcomes. However, more research may be necessary to understand how earlier engagement with lower-level operational managers and functionaries can be used to facilitate the uptake and sustainability of HSS interventions.

## References

[1] Rispel LC, Blaauw D, Ditlopo F, White J, Rispel LC, Padarath A (2018). Human resources for health and universal health coverage: progress, complexities and contestations. South African Health Review.

[2] van Rensburg HCJ (2014). South Africa's protracted struggle for equal distribution and equitable access – still not there. Hum Resour Health.

[3] van Rensburg HCJ, Engelbrecht MC, van Rensburg HCJ (2012). Transformation of the South African health system: post-1994. Heath and heath care in South Africa.

[4] Rispel L, Padarath A (2016). Analysing the progress and fault lines of health sector transformation in South Africa. South African Health Review.

[5] National Department of Health, Republic of South Africa (2011). Provincial guidelines for the implementation of the three streams of primary health care re-engineering.

[6] Pillay Y, Barron P The implementation of PHC re-engineering in South Africa.

[7] Free State Department of Health Free State Department of Health Ideal Clinic.

[8] Michel J, Chimbindi N, Mohlakoana N, Orgill M, Bärnighausen T, Obrist B (2019). How and why policy-practice gaps come about: A South African Universal Health Coverage context. J Glob Health.

[9] Malakoane B, Heunis JC, Chikobvu P, Kigozi NG, Kruger WH (2020). Public health system challenges in the Free State, South Africa: a situational appraisal to inform health systems strengthening. BMC Health Serv Res.

[10] World Health Organization (2008). Primary Health Care: Now More Than Ever.

[11] McEvoy R, Tierney E, MacFarlane A (2019). ‘Participation is integral’: understanding the levers and barriers to the implementation of community participation in primary healthcare: a qualitative study using normalisation process theory. BMC Health Serv Res.

[12] Republic of South Africa (2003). National Health Act 61 of 2003.

[13] Ngobeni V, Breitenbach MC, Aye GC (2020). Technical efficiency of provincial public healthcare in South Africa. Cost Eff Resour Alloc.

[14] World Health Organization (2018). Country cooperation strategy at a glance.

[15] Delobelle P, Wolhutte CC (2013). The health system in South Africa. Historical perspectives and current challenges. South Africa in Focus: Economic, Political and Social Issues.

[16] Statistics South Africa. How unequal is South Africa?.

[17] Mofolo N, Heunis C, Kigozi NG (2019). Towards National Health Insurance: Alignment of strategic human resources in South Africa. Afr J Prim Health Care Fam Med.

[18] Mayosi BM, Benatar SR (2014). Health and health care in South Africa – 20 years after Mandela. N Engl J Med.

[19] Coovadia H, Jewkes R, Barron P, Sanders D, McIntyre D (2009). The health and health system of South Africa: historical roots of current public health challenges. Lancet.

[20] Competition Commission South Africa (2018). Health market inquiry. Provisional findings and recommendations report.

[21] Zweigenthal VEM, Pick WM, London L (2019). Stakeholders' perspectives on Public Health Medicine in South Africa. PLOS ONE.

[22] Rispel L, Bruce J, Padarath A, King J, English R (2015). A profession in peril? Revitalising nursing in South Africa. South African Health Review 2014/15.

[23] McIntyre D, Ataguba J (2014). Access to quality health care in South Africa: Is the health sector contributing to addressing the inequality challenge? [Online].

[24] Booysen FlR (2003). Urban-rural inequalities in health care delivery in South Africa. Development Southern Africa.

[25] Gordon T, Booysen F, Mbonigaba J (2020). Socio-economic inequalities in the multiple dimensions of access to healthcare: the case of South Africa. BMC Public Health.

[26] Bhorat H, Mayet N, Pillay U (2013). A nation in search of jobs: Challenges in employment creation in the South African labour market and policy suggestions. State of the Nation. South Africa 2012-2013.

[27] World Bank Unemployment, total (% of total labor force) (modeled ILO estimate). Data.

[28] Younger DS (2016). Health Care in South Africa. Neuron Clin.

[29] Khemani S, Chaudhary S, Scot T (2022). Strengthening Public Health Systems. Policy Ideas from a Governance Perspective. Policy Research Working Paper 9220.

[30] Omukuti J, Barlow M, Giraudo ME, Lines T, Grugel J (2021). Systems thinking in COVID-19 recovery is urgently needed to deliver sustainable development for women and girls. Lancet Planet Health.

[31] Haynes A, Garvey K, Davidson S, Milat A (2020). What can policy-makers get out of systems thinking? Policy partners' experiences of a systems-focused research collaboration in preventive health. Int J Health Policy Manag.

[32] World Health Organization (2007). Everybody business: strengthening health systems to improve health outcomes. WHO's framework.

[33] Swanson RC, Atun R, Best A, Betigeri A, de Campos F, Chunharas S (2015). Strengthening health systems in low-income countries by enhancing organizational capacities and improving institutions. Glob Health.

[34] de Savigny D, Adam T (2009). Systems thinking for health systems strengthening.

[35] Schneider H, English R, Tabana H, Padayachee T, Orgill M (2014). Whole-system change: case study of factors facilitating early implementation of a primary health care reform in a South African province. BMC Health Serv Res.

[36] George A, Olivier J, Glandon D, Kapilashrami A, Gilson L (2019). Health systems for all in the SDG era: key reflections based on the Liverpool statement for the fifth global symposium on health systems research. Health Policy Plan.

[37] Thomas P, McDonnell J, McCulloch J, While A, Bosanquet N, Ferlie E (2005). Increasing capacity for innovation in bureaucratic primary care organisations - a whole system participatory action research project. Ann Fam Med.

[38] Schneider H, van der Merwe M, Marutla B, Cupido J, Kauchali S (2019). The whole is more than the sum of the parts: establishing an enabling health system environment for reducing acute child malnutrition in a rural South African district. Health Policy Plan.

[39] Pritchett L, Sandefur J (2015). Learning from experiments when context matters. Am Econ Rev.

[40] Hawe P (2015). Lessons from complex interventions to improve health. Annu Rev Public Health.

[41] Bates MA, Glennester R (2017). The generalizability puzzle. Stanf Soc Innov Rev.

[42] Harrison R, Fischer S, Walpola RL, Chauhan A, Babalola T, Mears S (2021). Where do models for change management, improvement and implementation meet? A systematic review of the applications of change management models in healthcare. J Healthc Leaders.

[43] Free State Department of Health (2015). Policy on the Implementation of the Health Systems Governance and Accountability (HSGA) Model.

[44] Kaplan RS, Norton DP (1992). The Balanced Scorecard - measures that drive performance. Harv Bus Rev.

[45] Malakoane B (2022). The Health Systems Governance and Accountability model for health service integration and performance improvement in the Free State.

[46] StataCorp. (2019). Stata Statistical Software: Release 16.

[47] Jørgensen KB, Jensen LE (2011). Introduction to NVivo 9.0.

[48] Thwala SBP, Blaauw D, Ssengooba F (2018). Measuring the preparedness of health facilities to deliver emergency obstetric care in a South African district. PLOS ONE.

[49] Kemppainen JK (2000). The critical incident technique and nursing care quality research. J Adv Nurs.

[50] Applebaum SH, Wohl L (2000). Transformation or change: Some prescriptions for health care organizations. J Serv Theory Pract.

[51] Republic of South Africa (1999). Public Finance Management Act, No. 1 of 1999. Government Gazette.

[52] National Treasury, Republic of South Africa (2001). Treasury Regulations for departments, [and] constitutional institutions, public entities, Parliament and provincial legislatures. Government Gazette.

[53] National Treasury, Republic of South Africa (2000). Treasury Regulations for departments, constitutional institutions and trading entities. Government Gazette.

[54] Atim C, Bhushan I, Blecher M, Gandham R, Rajan V, Davén J (2021). Health financing reforms for Universal Health Coverage in five emerging economies. J Glob Health.

[55] Provincial Government of South Africa Free State Department: Health. Financial.

[56] Kabene SM, Orchard C, Howard JM, Soriano MA, Leduc R (2006). The importance of human resources management in health care: a global context. Hum Resour Health.

[57] Schwartz RW, Tumblin TF (2002). The power of servant leadership to transform health care organizations in the 21st-century economy. Arch Surg.

[58] Department of Health, South Africa (1997). White Paper for the Transformation of the Health System in South Africa. Notice 667.

[59] Toor SR, Ogunlana SO (2009). Ineffective leadership: Investigating the negative attributes of leaders and organizational neutralizers. Eng Constr Archit Manag.

[60] Halbert RJ, Kabene SM, Orchard C, Howard JM, Soriano MA, Leduc R (1998). Competencies for population-based clinical managers: A survey of managed health care medical directors. Am J Prev Med.

[61] Schaay N, Heywood A, Lehmann U, Ntuli A (1998). A review of health management training in the public health sector in South Africa. South African Health Review.

[62] National Department of Health, South Africa (2006). A National Human Resource Plan for Health.

[63] Econex The role of primary healthcare in health reform.

[64] Dookie S, Singh S (2012). Primary health services at district level in South Africa: a critique of the primary health care approach. BMC Fam Pract.

[65] Muzammil S, Lopes G (2021). Lack of primary health care services during pandemic: An urgent reminder!. J Fam Med Dis Prev.

[66] Ritshidze Free State. State of health.

[67] Gilson L, Pienaar D, Brady L, Hawkridge A, Naledi T, Vallabhjee K (2017). Development of the health system in the Western Cape: experiences since 1994. A Padarath and P Barron. [ed.]. South African Health Review 2017.

[68] Hove J, D'Ambruoso L, Kahn K, Witter S, van der Merwe M, Mabethaa D (2022). Lessons from community participation in primary health care and water resource governance in South Africa: a narrative review.

[69] Haricharan HJ, Stuttaford M, London L (2021). The role of community participation in primary health care: practices of South African health committees. Primary Health Care Research & Development.

[70] Luxon L (2015). Infrastructure – the key to healthcare improvement. Future Hosp J.

[71] Maphumulo WT, Bhengu BR (2019). Challenges of quality improvement in the healthcare of South Africa post-apartheid: A critical review. Curationis.

[72] Mochoari R Covid-19: Free State in third wave with old problems, health workers say.

[73] Rao GN (2002). How can we improve patient care?. Community Eye Health.

[74] Williamson L, Stoops N, Heywood A (2001). Developing a District Health Information System in South Africa: A social process or technical solution?. MEDINFO 2001.

[75] Institute of Medicine (2001). Crossing the quality chasm: a new health system for the 21st century.

[76] Garrib A, D'Ambruoso L, Kahn K, Witter S, van der Merwe M, Mabethaa D (2008). An evaluation of the District Health Information System in rural South Africa. S Afr Med J.

[77] Haux R (2006). Health information systems – past, present, future. Int J Med Inform.

[78] Almunawar MH, Anshari M, Younis MZ, Kisa A (2015). Electronic health object: Transforming health care systems from static to interactive and extensible. Inquiry.

[79] Ball MJ, Peterson H, Douglas JV (1999). The computerized patient record: a global view. M.D. Computing: Computers in Medical Practice.

[80] Byson LF, Honey M (2021). Data entry form designing tools and software usability in DHIS2. Nurses and Midwives in the Digital Age. Selected Papers, Posters and Panels from the 15th International Congress in Nursing Informatics.

[81] Muhoza P, Tine R, Faye A, Gaye I, Zeger SL, Diaw A (2022). A data quality assessment of the first four years of malaria reporting in the Senegal DHIS2, 2014-2017. BMC Health Serv Res.

[82] Bhattacharya AA, Umar N, Audu A, Felix H, Allen E, Schellenberg JRM (2019). Quality of routine facility data for monitoring priority maternal and newborn indicators in DHIS2: A case study from Gombe State, Nigeria. PLOS ONE.

[83] World Health Organization Referral Systems - a summary of key processes to guide health services managers.

[84] MEASURE Evaluation Referral Systems Assessment and Monitoring Kit. PEPFAR, USAID, MEASURE Evaluation, 2013.

